# The feasibility and acceptability of an inoculative intervention video for gambling advertising: a focus group study of academics and experts-by-experience

**DOI:** 10.1093/pubmed/fdae167

**Published:** 2024-08-05

**Authors:** Jamie Torrance, Conor Heath, Marie O’Hanrahan, Philip Newall

**Affiliations:** School of Psychology, Swansea University, Swansea SA2 8PP, UK; School of Psychology, University of Chester, Chester CH1 4BJ UK; Faculty of Computing, Engineering, and the Built Environment, Birmingham City University, Birmingham B4 7AP UK; School of Psychology, Cardiff University, Cardiff CF10 3AT UK; School of Psychological Science, University of Bristol, Bristol BS8 1TU UK

**Keywords:** focus groups, gambling advertising, gambling marketing, inoculation, lived experience

## Abstract

**Background:**

Gambling advertising employs a range of persuasive strategies. We therefore aimed to evaluate a counter-advertising intervention video to increase resilience to gambling advertising persuasion.

**Methods:**

Three in-depth focus groups were conducted, and each group contained a mixture of gambling-related academics (*N* = 12) and experts with lived experience of gambling-related harm (*N* = 10). Participants were given access to the intervention video and provided feedback during the focus groups. Qualitative data were audio recorded and thematically analysed by the research team.

**Results:**

Three main themes were identified. First, participants recommended a shorter video that had a simplified and digestible structure. Second, frequent real-world examples of gambling advertisements within the video were discouraged, and the inclusion of a relatable human voiceover was considered imperative to the receptiveness of the video. Finally, participants deemed it important to deliver psychologically grounded yet jargon-free content via a conversational style. An overall narrative framed by consumer-protection was also preferred in order to increase acceptance of the video content, rather than a more didactic framing.

**Conclusions:**

Evaluating the acceptability of a counter advertising intervention video provided valuable insight from both an academic and lived-experience perspective. Such insight is instrumental to the meaningful co-design of counter-advertising interventions.

## Introduction

Gambling advertising is extensively financed and pervasive in jurisdictions that have liberalized gambling. For example, the US, UK and Australian gambling industries collectively spend approximately $4 billion (USD) on gambling advertising each year.^1–3^ These advertisements take many forms, including standalone television commercials, sports sponsorship, social media posts by operators and direct texts/emails to consumers.^4–6^ While some jurisdictions have imposed bans or stringent controls on gambling advertising to protect public health, other jurisdictions, notably the UK, have adopted a more lenient approach, permitting the gambling industry to largely self-regulate its advertising practices.^7^ Relatedly, consumers in these jurisdictions are often critical of this approach and commonly describe their daily experiences as being ‘saturated’ with gambling advertisements.^8–10^

Gambling advertising strategies are designed to be highly persuasive, often depicting gambling in a positive light with an exclusive focus on wins rather than losses and targeting specific demographics through tailored content.^4^^,^^5^^,^^11^^,^^12^ The gambling industry also frequently incorporates its logos into sporting events, which can exploit fans’ emotional ties to the sport, thereby enhancing the perceived legitimacy and recall of the gambling brand.^13–16^ These gambling logos are ubiquitous in UK sports, for example, with an average of 1565 gambling logos observed per English Premier League football match during the 2022/2023 season.^17^ Additionally, financial incentives/inducements like ‘free bets’ and ‘sign-up bonuses’ are widely advertised by the gambling industry, and despite their complex terms, can encourage riskier bets.^18–22^ Affiliate marketing further extends the reach of these strategies, with commissioned third-parties promoting bets often under the guise of expert advice via social media, despite potential conflicts of interest and the low success rates of these promoted bets.^23–25^

Very few measures have been implemented to address these commercially manipulative strategies used by the gambling industry. In the UK, regulatory interventions have mandated the inclusion of harm-reductive messages in gambling advertisements, such as the slogans ‘When the FUN stops, stop’ and ‘Take time to think’. However, these industry-favoured slogans show no evidence of providing a protective effect on gambling behaviour,^26^^,^^27^ and have been criticized for emphasizing personal responsibility rather than addressing the broader determinants of gambling harm.^28^ The UK Department for Culture, Media and Sport (DCMS) plans to introduce independently designed safer gambling messages by mid-2024 to optimize the effectiveness of these messages. Although this approach is warranted,^29^ field-studies suggest that safer gambling messages may have minimal impact on gambling behaviour,^30^ and such messages primarily focus on general gambling harms rather than fostering resilience to gambling advertising itself.

The recently launched ‘Odds are, they win’ campaign represents a new and independent approach by educating the public about gambling harms through social marketing without emphasizing personal responsibility.^31^ However, this campaign does not primarily focus on inoculating the public against gambling advertising strategies, and its overall efficacy remains untested. Consequently, there is a need for independent counter-advertising interventions that equip consumers with the knowledge to make more informed and self-directed choices in response to gambling advertising. Such interventions should also aim to cultivate a critical view towards the credibility of gambling advertisements to encourage future resilience. This approach has proven efficacious in building resilience among consumers against the persuasive advertising strategies of the alcohol^32^^,^^33^ and tobacco industries.^34–36^ However, to the best of our knowledge, such an intervention has not been developed and empirically tested in relation to gambling advertising.

In line with practices common within the fields of health and medicine, it is imperative to first evaluate the feasibility and acceptability of counter-advertising interventions prior to their implementation.^37^ This approach can help researchers prepare for more definitive and empirical testing of interventions (often through randomized controlled trials; RCTs) by highlighting areas in need of improvement without wasting valuable resources.^38^ A practical and economic method of attaining this insight involves the use of in-depth qualitative focus groups, providing valuable feedback from the perspectives of potential users and subject experts.^39^ In the context of developing interventions to counteract the influence of gambling advertising, it is important to consider the perspectives of both academic experts and Experts by Experience (EbyE). While academics can provide useful research-based insights, EbyE, who have been impacted by gambling-related harm, can offer their lived experiences to ensure that interventions are effectively grounded.^40^^,^^41^ However, to date there has been limited research involving the perspectives of EbyE in the co-design and development of gambling-related interventions.

There is a need for counter advertising interventions that are developed independently of the gambling industry. Before implementation, the feasibility and acceptability of such interventions should be evaluated with input from those with experience of gambling-related harm. Consequently, via the use of focus groups, here we will evaluate a brief intervention video aimed at fostering resilience against gambling advertising persuasion using a sample of academics and EbyE. The current study is therefore guided by two research questions:

(1) What are the perceptions and opinions of academics and EbyE regarding the acceptability and feasibility of the intervention video?

(2) According to these individuals, how can the intervention video be improved or developed before it is empirically tested via RCT?

## Methods

The focus groups were preregistered (https://osf.io/x5rwe), with the raw qualitative data (redacted where necessary) and materials available from https://osf.io/bv89u/. Ethical approval for the focus groups was obtained from the University of Chester and University Centre Shrewsbury ethics board.

### Intervention video

The focus groups were centred around the evaluation of a prototype intervention video that aimed to increase resilience against gambling advertising strategies. The video content was conceptually framed by ‘inoculation theory’,^42^ which proposes attitudinal resilience to persuasion can be fostered by controlled exposure to ‘weakened’ forms of that persuasion. In the context of the current study, this process involved depicting real-world examples of gambling advertising strategies to highlight the associated ‘threat’ of commercial manipulation. Next, ‘refutational pre-emption’ was encouraged by providing participants with potential counterarguments to this ‘threat’ and subsequently offering logical refutations to these counterarguments.^42–44^ In relation to gambling advertising, an example ‘threat’ component within the intervention video involved the message: ‘Financial incentives offered within gambling advertisements (such as free bets) can encourage more impulsive and risky betting decisions’. The counterargument to this ‘threat’ was: ‘It is the consumer’s choice to take up these incentives, and they are a free opportunity to win more money’. Finally, a logical refutation to this counterargument was: ‘These incentives are psychologically alluring, and are often subject to strict conditions that are difficult to interpret. These conditions significantly limit the likelihood of being able to redeem any tangible winnings’.

In combination, highlighting ‘threats’ and the encouragement of refutational pre-emption can build resilience to future persuasive attempts found in advertising strategies.^45–47^ This inoculative approach often also provides ‘umbrella protection’ against strategies that were not included in the initial intervention.^43^ The prototype intervention video within the current study covered five key gambling advertising strategies based on two previously conducted reviews.^4^^,^^5^ Specifically, these strategies included: (i) financial incentives; (ii) the promotion of risky bets; (iii) promotion in sports; (iv) affiliate marketing and (v) targeted advertising. The intervention video provided a ‘threat-counterargument-refutation’ segment for each of these five advertising strategies,^43^ which were accompanied by visual examples. The total runtime of the prototype intervention video was 11 minutes, and it was hosted on YouTube in an unlisted format to avoid public access at this stage.

### Participants and recruitment

A purposive sample of gambling-related academics (*n* = 12) and EbyE (*n* = 10) were recruited via email between May and July 2023. The inclusion of these groups was underpinned by the need to embed lived-experience and professional insight into the intervention design.^48^ Data collection was discontinued once a valid and robust understanding of the phenomena had been attained, thereby reaching data saturation.^49^ Following an initial information sheet, participants consented digitally and provided brief demographic information before the focus groups commenced (see Table 1). All participants were ethically compensated as consultants and therefore each received £150 for their contributions within the focus groups.^50^

**Table 1:** Demographic characteristics of the sample

Demographic category	Academics: N (%)	Ebye: N (%)	Total: N (%)
**Age:** mean (SD)	40.5 (10.9)	44.4 (11.5)	42.3 (11.1)
**Years of experience:** mean (SD)	13.8 (7.3)	17 (12.6)	15.2 (9.9)
**Sex**			
Male	7 (31.8)	5 (22.7)	12 (54.5)
Female	5 (22.7)	5 (22.7)	10 (45.5)
**Ethnicity**			
White	10 (45.5)	9 (40.9)	19 (86.4)
Black	0 (0)	1 (4.5)	1 (4.5)
Turkish	1 (4.5)	0 (0)	1 (4.5)
Australian	1 (4.5)	0 (0)	1 (4.5)
**Residing region**			
UK	10 (45.5)	8 (36.4)	18 (81.8)
Ireland	0 (0)	2 (9.1)	2 (9.1)
Australia	2 (9.1)	0 (0)	2 (9.1)

### Procedure

Three focus groups were conducted over Microsoft Teams with each containing 6–9 participants consistent with the recommendations made by Gill, Stewart^51^ as well as Stewart and Shamdasani.^52^ Each focus group consisted of both academics and EbyE in order to facilitate diverse perspectives and collaborative discussions.^53^ We utilized a semi-structured discussion guide developed through an iterative process. This involved analysing data from transcripts and researchers’ notes obtained during the initial focus group, which then informed and guided the discussions in subsequent sessions.^54^ However, all focus groups broadly addressed: (i) opinions towards the process of gambling advertising persuasion inoculation; (ii) the feasibility and acceptability of the intervention video; (iii) areas of strength and (iv) areas in need of improvement. Prior to each focus group, participants were sent a link to the intervention video and were instructed to make personal notes based on their perceptions. At the start of each focus group, participants gave permission for audio recording for the sake of transcription and analysis. JT led the focus groups with the assistance of CH, who took shorthand notes of important and relevant discussion points throughout. The focus groups lasted between 75 and 93 minutes, and all participants contributed substantially.

### Analysis

The audio data from the focus groups were transcribed verbatim and thematically analysed via an inductive approach. Our analyses were informed by the steps provided by Braun and Clarke.^55^ These analyses were conducted to establish underlying themes and patterns relating to the participants’ perceptions and opinions of the intervention video. JT engaged with initial data familiarization and immersion before inductively highlighting codes derived from key concepts that were pertinent to the research questions. JT, CH and MOH subsequently sorted these codes into higher-order preliminary themes based on their connectedness and salience. Regular meetings involving the research team were then conducted in order to refine these preliminary themes to produce and name more distinctive main themes. The final main themes were solidified through discussions by the research team to ensure their relevance and workability.^56^

## Results

Our analyses resulted in three main themes relating to participants’ perceptions and opinions towards the intervention video. These included its: 1) structure and length, 2) aesthetics and delivery and (iii) language and narrative. Our interpretations of these themes are discussed below with reference to corresponding participant quotes.

### Theme one: structure and length

The intervention video was comprised of three separate elements that covered the ethical issues surrounding gambling advertising strategies, why scepticism is important in relation to such advertisements, and an inoculative segment for each advertising strategy. Although positive feedback was provided, participants also expressed that this structure should be ‘simplified’ to improve the ‘flow’ of the video. Combining the elements in a more cohesive manner was encouraged by participants across all focus groups. This recommendation also stemmed from the overuse of text within the video, which was perceived as contrary to popular short-form and visually engaging content found on platforms such as TikTok. As a result, it was suggested that the video be presented in a more concise and impactful way.

*Everything now is just so quick and transactional, so you got to just be punchy* (Participant 22, Expert by experience)

Similarly, the video length of 11 minutes received critical feedback as it was deemed imperative to ensure that the audience does not ‘switch off’ while watching. Participants stated that this was particularly important if targeted towards young people.

*I think it was hard to follow and for younger people they are also potentially struggling a bit with longer attention. So over 10 minutes, it might be quite hard to follow that* (Participant 5, Gambling-related academic)

Overall, a more digestible and succinct video was suggested by participants in order to grab the audience’s attention. A common recommendation in order to achieve this aim involved incorporating a contents summary at the start of the video. This initial ‘hook’ was considered easy to implement and would allow the audience to quickly understand the context and aims of the video within a few seconds of watching.

*I thought it might be useful to have a really brief statement upfront about “these are the topics that we’re going to cover” and “what are the learning objectives”...I think that would take, probably ten seconds to say, but to just give a little bit of a road map to people about where the video is going* (Participant 17, Gambling-related academic)

### Theme two: aesthetics and delivery

Although visible examples of gambling advertising and logos within the video were recommended where necessary, their over-use was discouraged by participants. It was expressed that the abundance of gambling advertising examples within the video may actually be contrary to its intended aims due to unnecessary ‘exposure’ that was not deemed ‘appropriate’. Instead, it was expressed that more sparingly providing real examples of gambling advertising strategies alongside comparisons to the tobacco or alcohol industry would highlight the well-established ‘playbook’ of persuasive marketing practices.

*So maybe one of the ways to think about overcoming this problem of having rolling gambling adverts is to even frame it in the context of ‘look, this potentially harmful product is being marketed to you in the same way that all harmful products have been marketed in the past’.. ‘look at this advert for Marlborough cigarettes from 1974, with Ayrton Senna head to toe in Marlborough packets’.. then queue a gambling advert of Jose Mourinho in the back of a taxi.* (Participant 11, Gambling-related academic)

Given that the video was in a prototype state, certain segments contained stock footage to complement the audio voiceover. For example, rather than footage of a person specifically experiencing gambling harm, more generalized footage of someone expressing anger or frustration was used. However, EbyEs often stated that such footage lacked realism and created a ‘misalignment’ between the visual aesthetics and the overall message of the video.

The voiceover within the intervention video was generated via text-to-speech software. Participants perceived this ‘robotic’ voiceover to be ‘monotonous’ and expressed concern that it would reduce the receptiveness of the video.

*I think the voice, certainly I agree it was a little bit robotic. Personally, it annoyed me a little bit, but I think with an actual voice it could be improved* (Participant 13, Expert by experience)

It was therefore deemed essential that a professional narrator be utilized to ‘humanise’ the video in order to increase its receptiveness.

### Theme three: language and narrative

Some of the language utilized within the intervention video was often considered ‘overcomplicated’ and ‘too sophisticated’ for non-academic audiences to absorb. A more ‘conversational’ style that was free of jargon was proposed by participants in order to improve the clarity and applicability of the video. Although simplified language was encouraged, participants still deemed it important for elements of ‘psychology’ to be woven into the video narrative. The introduction of psychological content was considered helpful in allowing audiences to understand that marketing manipulation is common, and not a personal shortcoming on behalf of the individual.

*When people are actually given more [psychological] information in terms of how they are probably thinking, they are able make sense of it and find out ‘OK, well, maybe I’m not too crazy after all, maybe this kind of distortion and this kind of corruption, in terms of the hijacking of the brain, can happen to anyone that’s exposed to this kind of product or material’* (Participant 8, Expert by experience)

Participants also expressed that the video was delivered in a ‘lecture’ style, which at times could be perceived as being ‘preachy’. A more advantageous strategy of implementing a ‘consumer focused’ narrative was recommended, whereby the audience are offered useful information to protect themselves.

*I know you’ve talked about the tactics and strategies that advertisers use, and I thought that again the topics were spot on, but it just.. I thought.. needed to be more engaging about; ‘hey folks, this is what you need to be careful of’, ‘these are some of the tactics that are used’, ‘these are things that you could look out for’, ‘don’t be fooled by this’, ‘be a bit wary of that’* (Participant 17, Gambling-related academic)

Similarly, posing challenges to the audience rather than using a one-way didactic style was another suggestion made by participants. For example, when covering the positive framing of gambling within advertising, using questions, such as ‘is this really what gambling is like for you?’ rather than ‘this is not a truthful representation of gambling’ was recommended. This was considered a more useful method of fostering autonomy among the audience and further increasing the personal relevance of the video content overall.

## Discussion

### Main findings of this study

Few interventions have been established to foster resilience against the range of persuasive advertising strategies employed by the gambling industry.^4^^,^^5^^,^^31^ Prior to implementation, it is important to evaluate such interventions with input from professionals and those with lived experience of gambling harm.^37^^,^^40^^,^^41^ This focus group study contributes to understanding in this area, by thematically analysing the perceptions and opinions of academics and EbyEs. As a result, we identified three main themes that centred around the: (i) structure and length, (ii) aesthetics and delivery and (iii) language and narrative of a counter-advertising intervention video.

### What is already known on this topic

Participants valued a shorter video that incorporated a simplified structure. This feedback aligns with existing research suggesting that educational video content should minimize cognitive load to enhance learning and retention.^57^ Therefore, before further investigation via RCT, the intervention video will be shortened from 11 to a more ideal time of 6 minutes. Similarly, in line with participant feedback, a contents section will be incorporated into the video to encourage engagement. This is a common strategy employed within online video content and provides a roadmap for audiences to digest at the start of the video. Although some individuals (such as experienced gamblers) may be well-versed in gambling advertising strategies, advertising literacy is generally low across most populations.^58^^,^^59^ Therefore, optimizing the current intervention video so the complexities of gambling advertising are comprehendible is imperative to the inoculation process.

The overuse of real-world gambling advertising within the intervention video was discouraged by participants. Although the impacts of gambling advertising exposure have been recently contested by the UK DCMS,^60^ a wealth of studies have demonstrated a correlation between self-reported exposure to advertising and increased gambling.^22^^,^^61–63^ Based on participant feedback, the intervention video should only include examples of such advertising when totally necessary, and should be framed in conjunction with the well-established advertising strategies of the tobacco and alcohol industry.^64–66^ In order to humanize the delivery of this content, participants also encouraged the use of a professional voiceover rather than text-to-speech software. Human voice narration has been considered superior to synthetic voice by increasing retention and acceptability of educational content.^67^

Our findings also indicate that participants prefer counter advertising narratives that encourage informed and rational consumer behaviour in response to gambling advertising.^68^ This stands in direct opposition to typical gambling advertisements, whereby rationality is often inhibited by emotional appeals, complex T&Cs and unclear risk warnings.^69–71^ In the UK, consumer protection platforms, such as the ‘Money Saving Expert’, have become increasingly popular.^72^ Such platforms provide education to enhance financial literacy and to help consumers identify genuine versus deceptive financial advertisements.^73^ Based on the feedback provided by participants, this popular trend should also extend to gambling advertising, which can be both enticing and misleading.^6^ Furthermore, counter-advertising interventions that emphasize consumer protection are contrary to the industry-favoured discourse of ‘responsible gambling’.^74^ By encouraging consumers to recognize and ultimately resist persuasive gambling advertising strategies, the emphasis is shifted away from individual responsibility and towards the commercial determinants of harm.^75^

Participants also emphasized the importance of delivering counter-advertising content without using ‘preachy’ or didactive language, which is less effective in educational or harm-reduction contexts.^76^^,^^77^ For instance, research within the field of substance misuse has consistently shown that merely emphasizing the associated negative consequences does not effectively deter individuals.^78^ Instead, a more beneficial approach involves equipping individuals with comprehensive knowledge that enables them to make well-informed decisions and fosters a sense of personal autonomy.^79^ In the current context, this approach extends beyond didactic and vague slogans, such as ‘Take time to think’. Rather, it offers consumers a more collaborative and empowering experience to help them discern the credibility of gambling advertising.

### What this study adds

This study demonstrates the importance of developing and evaluating gambling interventions independently of the industry, which may be reticent to support strategies that are ‘too successful’ in undermining their commercial efforts.^80^ However, recent research has demonstrated that independent design alone may not be sufficient to guarantee effectiveness.^81^ As we demonstrate here, the input of EbyE can provide additional insight to ensure that gambling-related interventions are appropriate and fit for purpose.^40^^,^^41^ We therefore recommend the increased involvement of EbyE within the co-design of gambling-related interventions and public health strategies as a future research priority. In the current context, given that tobacco and alcohol counter advertising interventions have been commonly adopted, similar approaches for gambling advertising represent a simple paradigm as part of a wider public health approach. Consequently, we also recommend prioritizing empirical research to explore the behavioural effects of such interventions and expanding the use of video-based inoculation strategies to other areas of gambling, particularly those involving potentially harmful product design features.^82^^,^^83^ Based on our findings, we recommend that these inoculation strategies should be succinct, jargon-free and should avoid the overuse of real-world gambling stimuli.

### Limitations of this study

This study has various limitations. First, a purposive sample of academics and EbyE were recruited. While we successfully gathered a sufficiently sized and diverse group for this focus group study, our findings may not fully reflect the broader perspectives and opinions of these particular groups. Second, by leveraging interpersonal communication and group dynamics, focus groups represent a useful method of intervention evaluation. However, participants in focus groups may encounter specific challenges, such as hesitancy to broach sensitive subjects in a communal environment, or susceptibility to common issues like response bias or groupthink.^84^ Although we aimed to minimise these issues by respectfully prompting participants to contribute where necessary, they are a common limitation of focus groups. Third, participants provided feedback relating to currently utilized gambling advertising strategies addressed within the intervention video. Gambling advertising strategies evolve quickly,^4^ and as such the intervention video may need to be regularly updated. However, successful inoculation creates an umbrella protection that is capable of building resilience against both persuasive attempts addressed and not addressed by the intervention.^43^^,^^85^

## Conclusions

This study provides valuable insight into the evaluation of a brief intervention video that aims to foster resilience against gambling advertising. This insight was provided via an academic and lived-experience perspective, which is instrumental to the meaningful co-design and development of gambling-related interventions. Subsequently, the evaluative feedback provided will be implemented into the intervention video before it is empirically tested via RCT. Findings from the focus groups also have implications for others developing similar interventions to counter gambling advertising persuasion. These interventions could be employed by harm reduction organisations or integrated into educational settings for widespread adoption and impact.

## Data Availability

Links to the data and materials underlying this research are available within the methods section of this article.

## Notes

**Jamie Torrance**, Lecturer in Psychology

**Conor Heath**, Research Assistant

**Marie O'Hanrahan**, Lecturer in Psychology

**Philip Newall**, Lecturer in Psychology
